# Evacuation of axillary hematoma causing brachial plexus compression in newly diagnosed leukemia: a case report and literature review

**DOI:** 10.1080/23320885.2026.2636313

**Published:** 2026-02-23

**Authors:** Isaac Mordukhovich, Hafsa Omer Sulaiman, Sean Frisbie, Anthony Tufaro, Bahar Bassiri Gharb, Antonio Rampazzo

**Affiliations:** a Department of Plastic Surgery, Cleveland Clinic, Cleveland, Ohio, USA; b Case Western Reserve University School of Medicine, Cleveland, Ohio, USA; c Department of General Surgery, Cleveland Clinic, Cleveland, Ohio, USA

**Keywords:** Brachial plexus, hematoma, leukemia, myeloproliferative neoplasm, compartment syndrome

## Abstract

Spontaneous soft tissue hematomas are recognized complications of myeloproliferative neoplasms but rarely implicate the brachial plexus. A large axillary hematoma could represent a surgical emergency due to compression of local neurovascular structures. We report a case of a 63-year-old male with a history of polycythemia vera who presented with an expansive axillary hematoma compressing his brachial plexus. The lesion’s profound size caused upper limb paralysis and exquisite pain on passive movment, raised suspicion for malignant hematologic progression, and provided a localized etiology for the patient’s plexopathy. Large-vessel compromise, central nervous system lesions, and soft tissue invasion were excluded by clinical evaluations and diagnostic imaging before the interdisciplinary decision was made to surgically evacuate the proven complex hematoma. The patient’s pain improved following decompression and another washout two days later. Concurrent hematologic workup identified acute myelomonocytic leukemia by bone marrow biopsy and initiated appropriate management. Twenty-four abstracts were screened from PubMed using keyword search terms and citation matching. Thirteen patients were identified, 4 of which had compartment syndrome symptoms secondary to hematoma in the setting of an undiagnosed hematological malignancy. All cases in the literature were concurrently diagnosed with chronic myeloid leukemia whereas the case we present was a manifestation of acute myeloid leukemia. Extensive mass effect from a deep tissue hematoma as the chief complaint for a patient with leukemic transformation of polycythemia vera is a rare presentation. An interdisciplinary approach with meticulous hematologic workup allowed for management of both, the lesion’s neuropathic mass effect and its underlying hematologic malignancy.

## Introduction

Myeloproliferative neoplasms (MPNs), such as polycythemia vera (PV), are associated with paradoxical thrombotic and hemorrhagic complications driven by acquired von Willebrand syndrome, platelet dysfunction, and endothelial abnormalities related to JAK2 mutations [1–3]. Leukemic transformation to acute myeloid leukemia (AML) occurs in approximately 3% of PV patients and portends a poor prognosis [4–6]. AML workup is typically prompted by symptoms of bone marrow failure, but extramedullary involvement is also a common initial finding in this subtype of leukemia, especially with myelomonocytic or monocytic differentiation [7–9]. However, presentation as a spontaneous compressive hematoma requiring urgent surgical intervention is exceedingly uncommon [10,11].

Brachial plexus compression from axillary hematoma is itself rare and most often reported in the context of trauma, anticoagulation, or iatrogenic vascular injury [12–14]. We present a rare intersection of these pathologies: leukemic transformation of JAK2-mutated, trisomy 8 PV manifesting as a spontaneous axillary hematoma causing acute brachial plexopathy.

## Methods

Verbal consent was obtained from the patient for permission to include his anonymized information in this case report. The review of literature was performed through PubMed on January 8, 2026. Records were obtained by free text input and Medical Subject Headings, with key search terms including (‘leukemia’ OR ‘myelodysplastic syndrome’) AND (‘compartment syndrome’ OR ‘hematoma’) AND ‘extremity’. English-language titles and abstracts were screened for relevance by two independent reviewers (I.M. and S.F.), with disagreements resolved by a third independent reviewer (A.R.). Additional literature was added through further review of relevant citations in the identified records. Extracted data included patient demographics, hematological history and lab values, history of extremity trauma and presentation, treatment method, and treatment outcomes. Records were excluded if they discussed hematomas or compartment syndrome in a location besides the limbs or if there was no evidence of malignancy found.

## Case report

**Figure 1.**
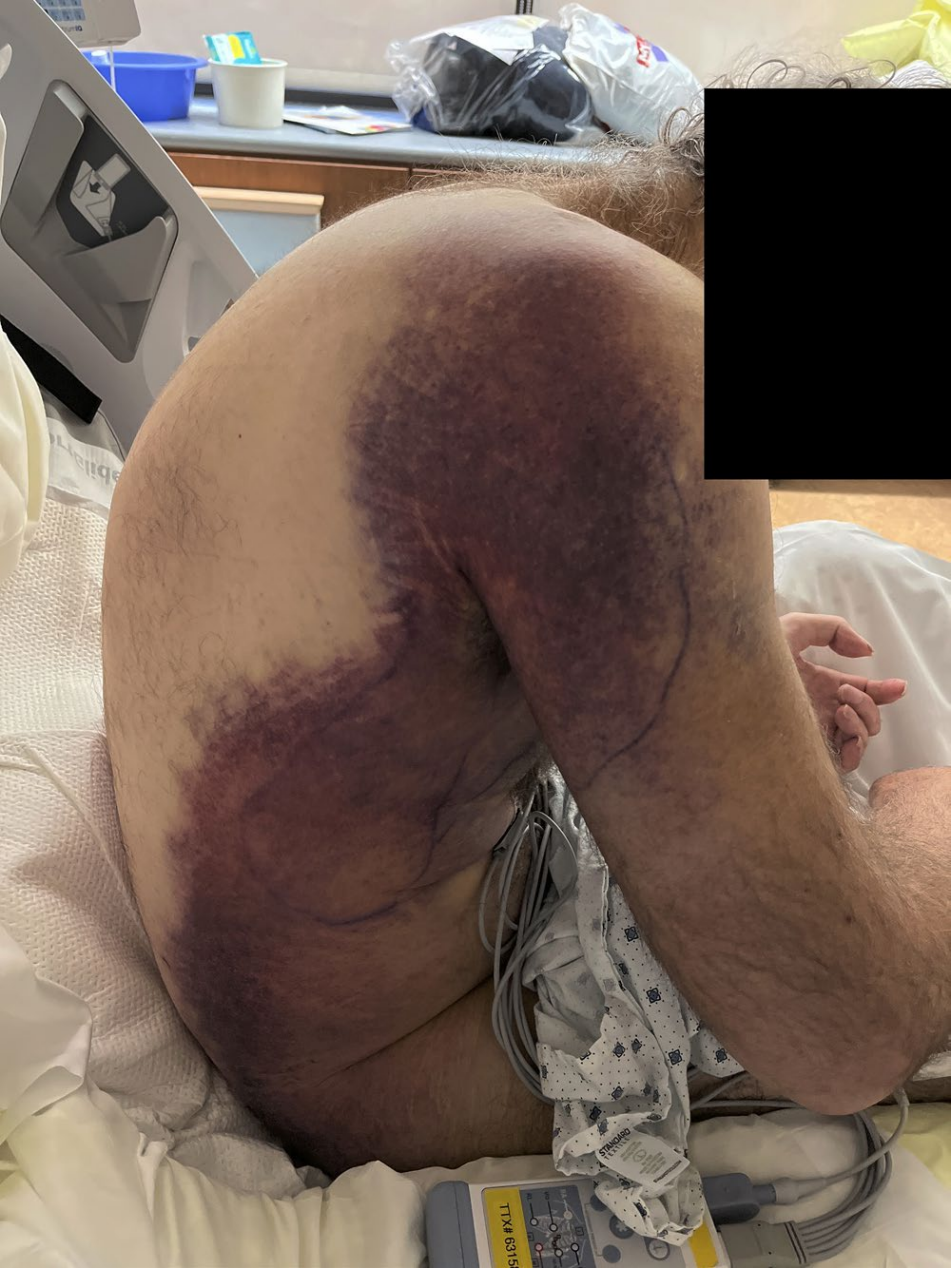
Preoperative photo demonstrating the extent of violaceous discoloration observed along the patient’s upper arm and flank.

A 63-year-old Caucasian male with a history of heavy former tobacco use, splenectomy, and JAK2+, trisomy 8 PV presented to a regional emergency department for worsening pain and loss of strength in his right shoulder after a 3-month history of intermittent pain. He had previously been undergoing regular therapeutic phlebotomy for his PV but was lost to follow-up 11 months prior due to financial strains. On exam, he had loss of shoulder flexion with preserved finger movement and a 7.3 cm x 6.7 cm, violaceous mass in his right axilla (Figure 1), which was found to be a 11.4 cm x 6.2 cm x 5.7 cm hematoma on computed tomography (CT). Laboratory work up revealed additional systemic complications to the patient’s presentation, including suspicions for malignant progression of his PV with resultant coagulopathy and acute kidney injury (secondary to tumor lysis syndrome).

**Figure 2.**
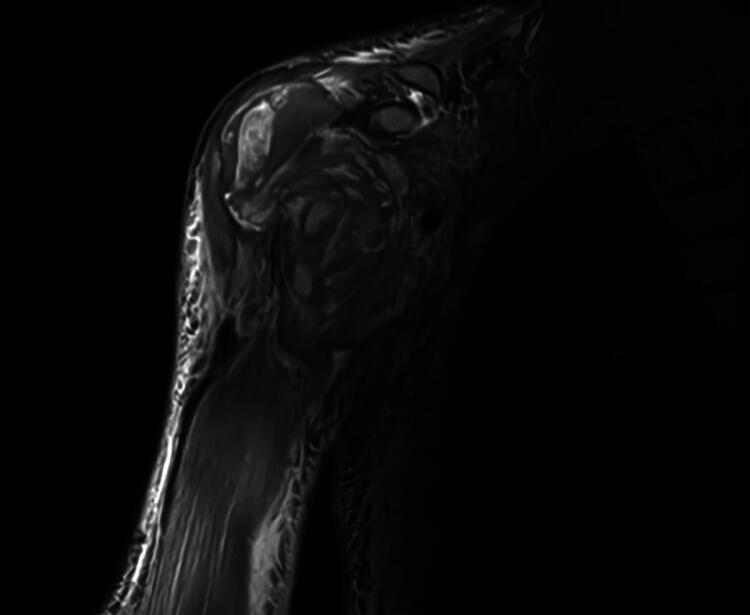
Preoperative MRI showing a heterogeneous, fluid-filled space-occupying lesion in the axilla.

The patient was transferred to the Cleveland Clinic for specialized management of the suspected hematologic malignancy. On admission, the patient’s axillary mass was promptly examined for compartment syndrome due to evidence of pain with passive movement and worsening paresis of the right arm, which now extended distal to the humerus along with accompanying paresthesia. Hematologic malignancy involvement of axillary nodes and brachial plexus with bleeding and brachial plexus compression by the spontaneous hematoma because of impaired coagulation were chief among our differentials, but the notable dissociation of nearly week-long, worsening neurologic deficits introduced suspicion for central nervous system causes. However, computed tomography of the head (CTH) found no deficits and instead, computed tomography angiography (CTA) and magnetic resonance imaging (MRI) of the chest wall and upper extremity confirmed mass effect compression of the brachial plexus and axillary vein by the complex hematoma in the setting of diffuse muscle and subcutaneous edema (Figure 2). No evidence of infiltrative soft tissue malignancy was found. With absence of an invasive pathology and continuing progression of the patient’s brachial plexus compression symptoms, an interdisciplinary recommendation was made to evacuate the hematoma.

Approximately 400 cc of fluid was evacuated from the axilla and medial upper arm upon drainage of the hematoma, which had extended along the course of the brachial plexus and stained the deep fascial compartments with blood. Diffuse oozing persisted at the conclusion of the surgery, so the wound was packed and closed proximally over the exposed axillary vein. The patient was then transferred to the intensive care unit (ICU) post-operatively due to vasopressor requirements in the setting of hypovolemic shock, which also necessitated 2 units of packed red blood cells and 1 unit of fresh, frozen plasma in the immediate post-operative period. He remained in the ICU uneventfully for 2 days before returning to the operating room for re-exploration, washout, and definitive closure with drain placement. Postoperatively, the patient experienced improvement in pain but persistent sensory loss and weakness of the right upper extremity, which is planned to be further managed with out-patient occupational therapy and follow-up to monitor nerve recovery.

A hematologic workup concurrent with management of the axillary hematoma revealed that the patient’s PV had transformed to acute myelomonocytic leukemia with KMT2A rearrangement (137,580 leukocytes/μL; 112,820 monocytes/μL; 4.0% blasts; 259,000 platelets/μL). A regimen of azacitidine and venetoclax was therefore initiated with plans for out-patient chemotherapy on discharge. Additional evaluation of the patient’s noted bleeding diathesis was performed, finding prolonged PT (17.9) and aPTT (51.6). Addition of vitamin K normalized the PT/INR, but aPTT continued to be elevated and did not correct on independent mixing studies. Together, these findings suggested a vitamin K deficiency and subclinical lupus anticoagulant presence, respectively. The former likely contributed to the extensive hematoma formed and the latter was confirmed on further lab testing.

## Review of literature

**Figure 3.**
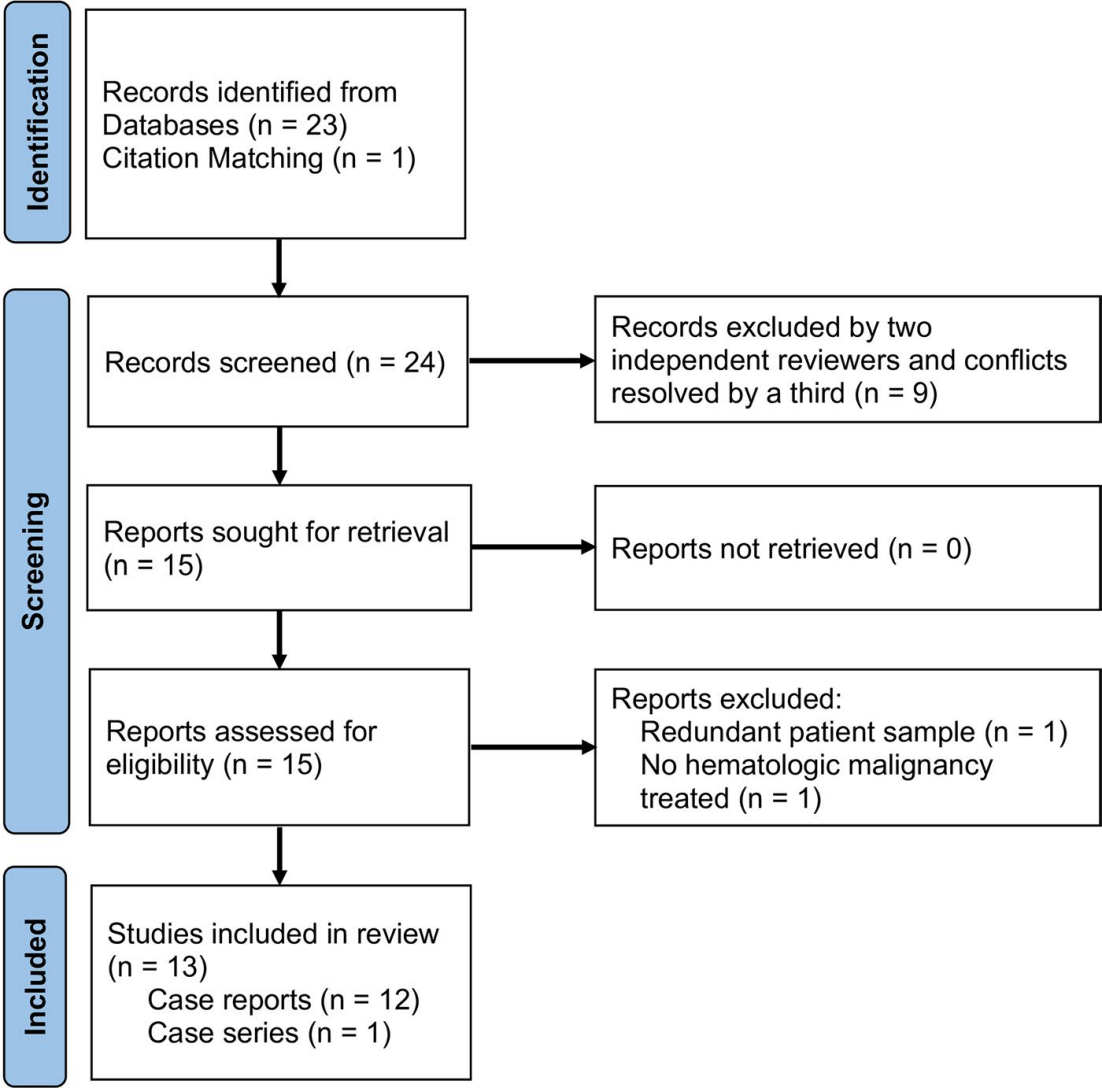
Preferred Reporting Items for Systematic reviews and Meta-Analyses diagram illustrating the workflow performed for the review of literature.

Thirteen of 24 papers were included (Figure 3), yielding 13 patients from 12 case reports and 1 case series (Table 1) [11,15–26]. One patient was included although further workup within the case report did not identify leukemia. However, the patient’s presenting lesion, myeloid sarcoma, was known to be heavily associated with AML and he was therefore still treated for cancer after surgical excision of the mass. Among all the leukemia diagnoses, hematomas were the most likely lesions to be found causing compartment syndrome in the extremities and only one was reportedly atraumatic (Nagase 2016). CML was the most common malignancy (7/13) and patients were predominantly male (6/7) and spanned ages 11–70 (median 49 [21.5–67.5] years) (Table 1). There is similarly no discernable trend in diagnostic methodology for the mass-occupying lesion in such instances, as detailed imaging (CT or MRI) and bedside methods (compartment pressure or ultrasound) were reported in 5 patients each. One patient was diagnosed with myeloid sarcoma only after pathological infiltrates were identified during fasciotomy, which were collected and sent for pathological evaluation. Hemorrhage (3/13) and persistent motor deficits (3/13) were common complications after compartment syndrome treatment. A statistically significant difference in complication type (major versus minor) between pediatric and adult patients could not be found in the present comparison (*p* = 0.266, Fisher’s exact test), likely due to low sample size.

**Table 1.** Summary of patients in reviewed literature.

Case	Author	Year	Gender	Age	Hx Leukemia	Hx MDS	Inciting Event	Location	Lesion	Skin Discoloration	Lesion Diagnosis	CS	Coagulopathy	PT	aPTT	Leukemia	Lesion Treatment	Re-Operation	Complications
1	Benhachem	2023	M	5	0	0	–	Thigh	Hematoma	0	US	0	Thrombocytopenia	WNL	WNL	AML	Platelet transfusion + antibiotics	NA	None
2	Cohen	2014	M	11	0	0	Incidental trauma	Calf	Hematoma	0	MRI	1	–	13.1	42	CML	Fasciotomy + surgical drainage	0	None
3	Lee	2011	M	11	0	0	Exercise	Thigh, Buttock	Hematoma-like	1	MRI	1	–	–	–	CML	Fasciotomy + drainage	0	Persistent motor weakness
4	Vajdic	2026	M	17	1	1	Atraumatic	Calf (bilateral)	None	0	–	1	–	–	–	B-ALL	Fasciotomy	0	None
5	Trumble	1987	M	20	1	1	Venous puncture	Forearm	Leukemic infiltrates	1	Compartment pressure	1	–	19.9	80.6	ALL	FFP + fasciotomy + cytosine arabinoside + radiation	0	AKI, pulmonary decompensation, and death due to metastasis
6	Scheipl	2007	M	30	0	0	–	Lower leg	Myeloid sarcoma	0	Pathology	1	–	–	–	AML*	Fasciotomy then idarubicin + cytosine arabinoside	0	None
7	Ng	2009	M	32	0	0	Minor trauma	Thigh	Hematoma	–	–	1	Platelet dysfunction	–	–	CML	Fasciotomy	0	Hemorrhage
8	D’Souza	2021	M	49	0	0	Exercise	Upper arm/axilla	Hematoma	1	CT+MRI	1	Platelet dysfunction	WNL	WNL	CML	Surgical drainage	1	Persistent motor weakness
9	Li	2025	F	51	1	1	Arterial puncture, ATRA + arsenic trioxide induction	Forearm	Hematoma	1	US	1	DIC	52.1	69.5	APL	Cold precipitate + FFP + hydroxyurea + dexamethasone + deoxyrubicin liposome	NA	Rhabdomyolysis, AKI, death
10	Hembd	2021	F	54	1	1	Central catheter placement, COVID-19	Upper arm	Phlegmasia Cerulea Dolens	0	US	1	Chemotherapy pancytopenia	–	–	Ph-like ALL	Heparin + fasciotomy + open CTR	0	Hemorrhagic shock, death (from COVID-19 respiratory compromsie)
11	Mangan & Luger	2012	M	65	1	1	–	Thigh	Hematoma	0	MRI	0	–	–	–	CML	Surgical drainage	0	None
12	Nagase	2016	F	72	0	0	Atraumatic	Forearm	Hematoma	0	CT	1	Platelet dysfunction	15.9	WNL	CML	Fasciotomy	0	Hemorrhagic shock
13	Quinn	2024	M	70	0	0	–	Forearm (bilateral)	None	1	Compartment pressure	1	Acquired factor 13 deficiency	42	WNL	CML	pRBC + fasciotomy + CTR + Guyon canal release	1	Altered sensation weakened grip (left > right)
**Present Case**		**2025**	**M**	**63**	**0**	**1**	**Atraumatic**	**Axilla**	**Hematoma**	**1**	**CT+MRI**	**1**	**Vitamin K** **Deficiency**	**17.9**	**51.6**	**AML**	**Surgical drainage**	**1**	**Persistent motor weakness**

CML was the most common diagnosis among patients with compartment syndrome or hematoma in the setting of hematological malignancy. Trends in age and gender within this group differed from that of the broader CML patient population. Within our review, the presented case is unique for its combination of location, atraumatic history, hematologic diagnosis, and presentation as the chief complaint resulting in diagnosis of AML. CS = compartment syndrome. NA = not applicable. WNL = within normal limits. – = not available. AML = acute myeloid leukemia. ALL = acute lymphoblastic leukemia. CML = chronic myeloid leukemia. Ph-like ALL = Philadelphia-like ALL. APL = acute promyelocytic leukemia. AML* = AML not proven on pathology, but suspected due to myeloid sarcoma pathophysiology. CTR = carpal tunnel release.

## Discussion

Hemorrhagic complications are less common than thrombotic events in MPNs, with reported prevalences of 8% and 54% in PV, respectively [27,28]. When bleeding does occur, it represents the presenting symptom in 3–8.1% cases of PV and involves the skin, mucous membranes or gastrointestinal tracts in approximately 52.9% of hemorrhagic events [29,30]. Bleeding outside of these sites is rare, but spontaneous subdural hematomas in patients with PV have been notably described in a limited number of case reports and systematic reviews [29,31,32]. Among atypical bleeding sites, involvement of the extremities is incredibly rare, composing fewer than 5% of reported hematomas in patents with PV and occurring almost exclusively in the lower limbs [11,24,29].

Spontaneous axillary hematoma causing brachial plexus compression is rare and most commonly reported in the setting of anticoagulation, trauma, or vascular intervention [7,10,33]. While no such trigger was identified in this patient, his severe leukocytosis (over 16,000/μL) in the setting of PV did place him at elevated risk for hemorrhage [34]. This was further exacerbated by MPN-associated hemostatic dysfunction and leukemic transformation-related coagulopathy [1–3].

Physical exam and bedside Duplex ultrasound findings supported the diagnosis of hematoma, but there remained concern for the lesion being an extramedullary soft tissue invasion from an underlying osseous neoplasm or the PV’s malignant transformation to AML. Extramedullary AML may directly cause neurologic symptoms by seeding the cerebrospinal fluid, developing into leptomeningeal disease or radicular nerve infiltration, or through the exceptionally rare invasion of peripheral nerves (neuroleukemiosis) [35]. A meticulous, interdisciplinary diagnostic plan between plastic surgery, thoracic surgery, neurosurgery, orthopedic oncology, and radiology was therefore executed to reduce suspicion for malignancy before any invasive actions were performed that could cause hematogenous seeding. CTH, CTA, and MRI findings all suggested a peripheral, compressive mechanism consistent with ischemic brachial plexopathy rather than direct nerve invasion.

The timing of surgical decompression is critical in cases of axillary sheath hematoma with neurologic compromise. Prior reports of axillary sheath hematomas demonstrate improved neurologic recovery with early evacuation, whereas delayed intervention increases the risk of permanent deficits [12,14,36]. Although further hematologic laboratory results could have provided a more definitive exclusion of malignancy, the situation was deemed a surgical emergency due to the patient’s rapidly deteriorating limb function and worsening pain. The patient was therefore counseled on his options before laboratory results were available and a shared decision was made to pursue surgical evacuation. Nonetheless, despite timely surgical management, the extent and prolonged pre-hospitalization progression of the hematoma likely resulted in irreversible ischemic nerve injury in this case, a complication also seen in multiple cases in existing literature. Aggressive management of the patient’s hemodynamic status resulted in rapid stabilization after he developed hypovolemic shock, which also occurred with multiple patients in our literature review.

The present case was unique among other patients in that it occurred without an inciting traumatic event, with a nutritionally acquired coagulopathy, and with an insidious AML pathology. All reported coagulopathies arose secondary to an underlying malignancy, while the presented patient’s coagulopathy was also nutritionally acquired. CML was the most common malignancy and reflected the disease’s greater incidence in males [37]. However, the patient ages did not reflect CML’s trends. The greatest incidence of CML in the United States 1975–2009 was in patients over 75 years old and only 24.6% of patients were 49 or younger [37]. In contrast, 49 was the median age for CML patients to develop compartment syndrome in the limb within the reviewed literature. Although a sample size of seven is insufficient to draw any conclusions, this finding may raise the clinical index of suspicion for evaluating hematological differentials in patients with minimally traumatic hematoma or compartment syndrome, especially with new-onset coagulopathy in younger individuals.

This case highlights a rare but high-risk surgical presentation in which hematologic malignancy manifests as an acute compressive neuropathy requiring urgent operative intervention. Surgeons should recognize spontaneous axillary hematoma as a potential surgical emergency in patients with myeloproliferative disease and collaborate with medical colleagues in acute and preventive management strategies for patients with MPN [38].

## Conclusion

Spontaneous axillary hematoma causing brachial plexus compression can represent the initial surgical manifestation of leukemic transformation in polycythemia vera or other myeloproliferative neoplasms. Although rare, early recognition and urgent decompression are critical to optimize neurologic outcomes while concurrent hematologic workup should be performed to evaluate for progression of hematologic malignancies.
